# Supplementary figures and images for: Nanosecond pulsed electric signals can affect electrostatic environment of proteins below the threshold of conformational effects: The case study of SOD1 with a molecular simulation study
Source: PLoS One. 2019 Aug 27;14(8):e0221685. doi: 10.1371/journal.pone.0221685 (PMC6711501; doi:10.1371/journal.pone.0221685)

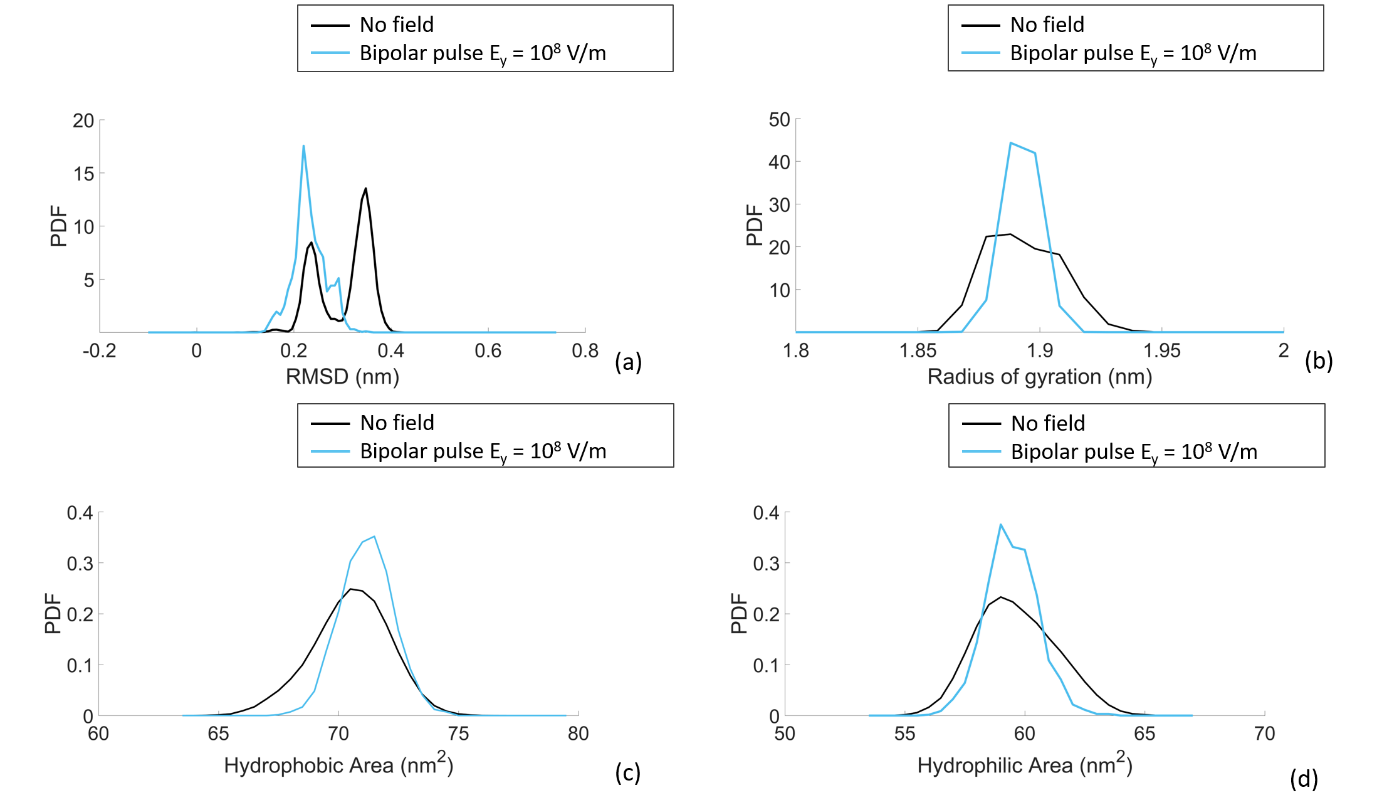

Supplement: S1 Fig — In the panel four different protein observables are presented as probability density distributions: the RMSD (A), the Radius of gyration (B) and the Hydrophobic (C) and Hydrophilic (D) areas. The curves refer to the protein structure in equilibrium condition (black line) and under the effect of external MP of 108 V/m intensity (light blue line). (TIF) [file pone.0221685.s001.tif]

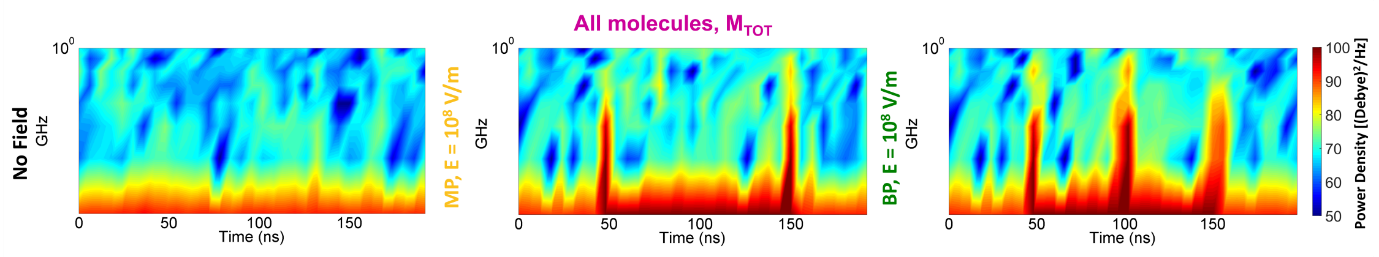

Supplement: S2 Fig — Spectrogram representation of the dipole moment (y-component) of all chemical species inside the simulation box, reported in absence of any exogenous field (black label) and in presence of a 100 ns, 108 V/m MP (yellow label) and BP (green label). (TIF) [file pone.0221685.s002.tif]

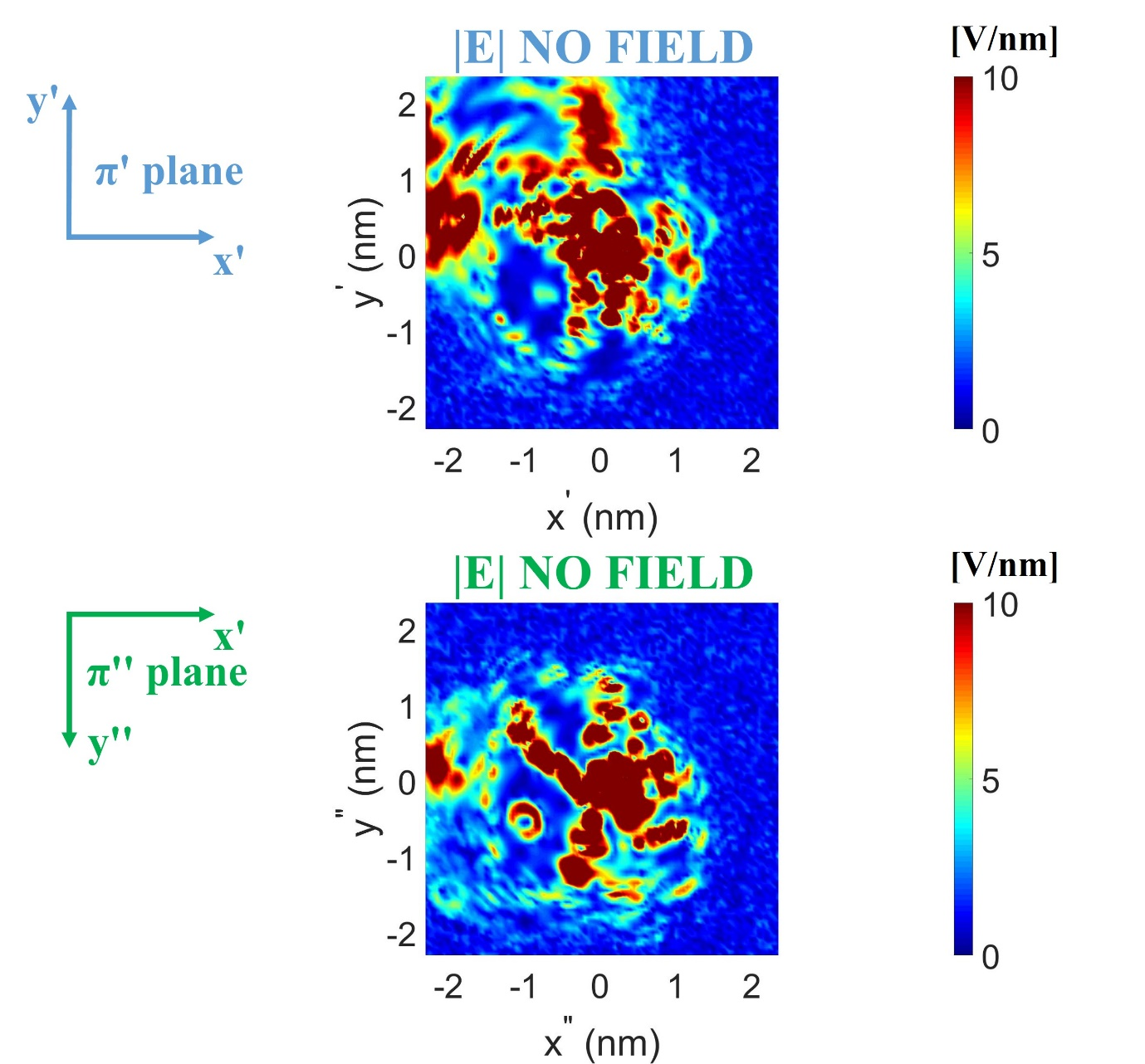

Supplement: S3 Fig — 2D-maps of the local electric field (absolute value depicted) around the active site on the π’ plane and the π” plane in the No field condition (first and second raw respectively). (TIF) [file pone.0221685.s003.tif]

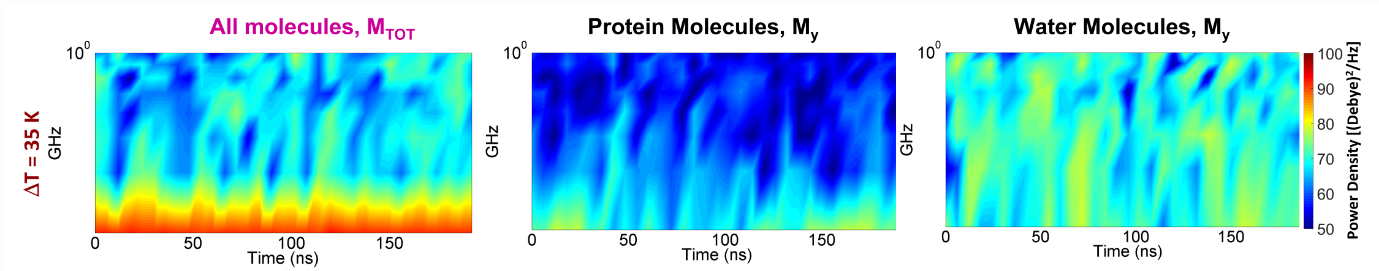

Supplement: S4 Fig — Effect of a 35K temperature increase on the frequency spectral content of the dipole moment (y-component) of all chemical species inside the simulation box (first column), the Cu,ZnSOD1 alone (second column) and the water molecules (third column). (TIF) [file pone.0221685.s004.tif]
